# The Use of Head-Worn Displays for Vital Sign Monitoring in Critical and Acute Care: Systematic Review

**DOI:** 10.2196/27165

**Published:** 2021-05-11

**Authors:** Francine Elrose, Andrew Hill, David Liu, Isaac Salisbury, Thien LeCong, Robert G Loeb, Penelope Sanderson

**Affiliations:** 1 School of Psychology The University of Queensland St Lucia, QLD Australia; 2 Minerals Industry Safety and Health Centre Sustainable Minerals Institute The University of Queensland St Lucia, QLD Australia; 3 Clinical Skills Development Service Metro North Hospital and Health Service Herston, QLD Australia; 4 School of Information Technology and Electrical Engineering The University of Queensland St Lucia, QLD Australia; 5 South Australian Ambulance Service (MedSTAR) Adelaide, SA Australia; 6 The Lyell McEwin Hospital Elizabeth Vale, SA Australia; 7 College of Medicine University of Florida Gainesville, FL United States; 8 School of Clinical Medicine The University of Queensland Herston, QLD Australia

**Keywords:** wearable, wearable device, head-mounted display, head-worn display, clinical setting, medical setting, patient monitoring, healthcare

## Abstract

**Background:**

Continuous monitoring of patient vital signs may improve patient outcomes. Head-worn displays (HWDs) can provide hands-free access to continuous vital sign information of patients in critical and acute care contexts and thus may reduce instances of unrecognized patient deterioration.

**Objective:**

The purpose of the study is to conduct a systematic review of the literature to evaluate clinical, surrogate, and process outcomes when clinicians use HWDs for continuous patient vital sign monitoring.

**Methods:**

The review was registered with PROSPERO (CRD42019119875) and followed the PRISMA (Preferred Reporting Items for Systematic Reviews and Meta-analyses) guidelines. A literature search was conducted for articles published between January 1995 and June 2020 using the following databases: PubMed, Embase, CINAHL, PsycINFO, and Web of Science. Overall, 2 reviewers independently screened titles and abstracts and then assessed the full text of the articles. Original research articles that evaluated the clinical, surrogate, or process outcomes of head-mounted displays for continuous vital sign monitoring in critical care or acute care contexts were included.

**Results:**

Of the 214 records obtained, 15 (7%) articles met the predefined criteria and were included in this review. Of the 15 studies, 7 (47%) took place in a clinical context, whereas the remainder took place in a simulation environment. In 100% (7/7) of the studies that evaluated gaze behavior, changes were found in gaze direction with HWDs. Change detection improvements were found in 67% (2/3) of the studies evaluating changes in the participants’ ability to detect changes in vital signs. Of the 10 studies assessing the ease of use of the HWD, most participants of 7 (70%) studies reported that the HWD was easy to use. In all 6 studies in which participants were asked if they would consider using the HWD in their practice, most participants responded positively, but they often suggested improvements on the HWD hardware or display design. Of the 7 studies conducted in clinical contexts, none reported any clinical outcomes.

**Conclusions:**

Although there is limited and sometimes conflicting evidence about the benefits of HWDs from certain surrogate and process outcomes, evidence for clinical outcomes is lacking. Recommendations are to employ user-centered design when developing HWDs, perform longitudinal studies, and seek clinical outcomes.

**Trial Registration:**

PROSPERO International Prospective Register of Systematic Reviews CRD42019119875; https://www.crd.york.ac.uk/prospero/display_record.php?RecordID=119875

## Introduction

### Background

Early recognition of patient deterioration can improve patient outcomes [1-3]. Vital sign monitoring helps clinicians track physiological parameters that can provide prodromal warning signs of critical illness [4]. In acute and critical care contexts, vital sign monitoring may reduce preventable in-hospital deaths by improving the early detection of clinical deterioration and allowing for timely intervention to reverse physiological decline [5-7].

There are two approaches to in-hospital vital sign monitoring: intermittent and continuous. Intermittent monitoring involves the measurement and recording of vital signs at regular time intervals (eg, 30 minutes). Some research suggests that some patient deterioration may not be detected because of the gap between observations [8]. Continuous monitoring, recognized as a more proactive approach that is typically used for more at-risk patients, involves the continuous capture of vital sign information or information about significant changes, which is then transmitted to a display device. Although the utility of continuous monitoring for low-intensity patients is still under debate, a recent systematic review focusing on general hospital wards found that continuous vital sign monitoring showed clinical benefits over intermittent monitoring, including reduced critical care use and reduced length of hospital stay, as well as an overall reduction in the cost of patient care [9].

One of the impediments to implement continuous vital sign monitoring is that it can increase the burden on health care workers if algorithms are not used to limit nonactionable or nuisance alarms [10]. The fact that current continuous vital sign monitoring systems have fixed physical locations—at the bedside or at a central station—might also increase the cognitive and physical workload of health care workers when they need to move to the monitor location to see patients’ status. Even when monitoring information is displayed nearby, it may be at a location that is awkward to see, such as located behind the anesthesiologist in the operating room. A solution may be the use of wearable devices to provide clinicians with continuous access to patient information, regardless of their location.

Head-worn displays (HWDs) are a type of wearable device that projects information in front of one eye (monocular) or both eyes (binocular) over a background that is either transparent or opaque. HWDs have been trialed in health care contexts for a variety of purposes, such as visual instruction and augmented reality during surgery, videoconferencing between physicians and consultants, and image and video recording for educational purposes [11].

HWDs offer several potential benefits in health care contexts. They allow clinicians to maintain sterility while accessing task-relevant information, which is beneficial in the clinical context [12]. They provide patient information independent of head orientation, which may be particularly advantageous when used for continuous patient monitoring. They present information that is *always there* while clinicians move around the environment and complete other tasks.

### Objectives

The purpose of this review is to examine the evidence for the effectiveness of HWDs for continuous patient vital sign monitoring in hospital or patient transport environments. Recent reviews have surveyed the broad range of uses of HWDs in surgery [11,13] or in a wide range of care situations [14]. Other reviews have focused specifically on Google Glass and its uses in surgical environments [15] or nonsurgical environments [16]. This review is not restricted to any specific HWD device. It also offers a more probing and detailed assessment of one specific class of use—the effect of HWD-based vital sign monitoring on outcomes—and it assesses the quality and risk of bias in the papers reviewed. Three types of outcomes are of interest: (1) clinical outcomes (measurable changes in mortality, morbidity, or patient complications), (2) surrogate measures (markers that may correlate with clinical outcomes but do not have a guaranteed relationship, such as detection of deterioration, or reduction in time to detect vital sign changes), and (3) process measures (procedural aspects related to the clinical process, such as changes in the pattern of gaze changes, or improvements in information sharing).

## Methods

### Overview

This systematic review was performed in accordance with the PRISMA (Preferred Reporting Items for Systematic Reviews and Meta-analyses) guidelines. The review was registered with the International Prospective Register of Systematic Reviews (CRD42019119875) before the search process started.

### Article Retrieval

We developed the search terms with the assistance of a professional librarian at The University of Queensland. The search strategy used keywords related to the concepts of *head-worn displays*, *vital signs*, and *patients*. The full search terms are included in Table S1 in Multimedia Appendix 1. The searches were run in the following databases in June 2019 and again in June 2020: PubMed, Embase, CINAHL, PsycINFO, and Web of Science. Search strategies were adapted for each database to allow for differences in the required search techniques. The search strategies are listed in Table S2 in Multimedia Appendix 1. Once the articles were selected, their reference lists were screened to identify eligible articles not located using the search strategy; these articles were then screened based on the same inclusion and exclusion criteria.

### Selection Criteria

The inclusion criteria were as follows: (1) peer-reviewed qualitative, quantitative, or mixed methods studies (excluding gray literature, editorials, systematic or other reviews, and meta-analyses); (2) real or simulated critical care or acute care clinical contexts; (3) fully trained clinician or clinical trainee participants; (4) HWDs used for vital sign monitoring (excluding images or videos without vital sign information); (5) studies with or without a comparator; and (6) predefined clinical, surrogate, or process outcomes. The list of outcomes was developed prospectively in collaboration with 2 practicing clinicians to focus on outcomes relevant to the clinical context and is provided in Table S3 in Multimedia Appendix 1. The full inclusion and exclusion criteria are also included in Table S4 in Multimedia Appendix 1.

### Data Extraction and Analysis

A standardized form was developed for the structured collection of data from each article, including publication details, study methodology, HWD details, and study outcomes; full details of the data collected are presented in Table S5 in Multimedia Appendix 1. For the study outcomes, classifications were made based on the descriptions of the outcomes by authors of the retrieved articles. Overall, 2 authors (FE and PS) independently extracted data from all the articles. Discrepancies were resolved through discussion with a third author (AH) when necessary. The data extracted from the articles were analyzed both quantitatively and qualitatively.

### Risk of Bias Assessment

The risk of bias assessment considers the extent to which the design of a study and methods used are likely to have prevented bias [17]. Two authors (FE and PS) independently assessed the risk of bias of each study using the Standard Quality Assessment Criteria for Evaluating Primary Research Papers from a Variety of Fields [18]. According to Kmet et al [18], each study is rated according to whether it meets specific criteria with *yes* (2 points), *partial* (1 point), or *no* (0 points), with a *not applicable* (“N/A”) option available for selected criteria. A summary score is then calculated across all relevant items as a percentage of the possible total score. A higher percentage represents an assessment of higher-quality research and lower risk of bias. Where a study uses both quantitative and qualitative methodologies, it is assessed against both criteria, and 2 summary scores are calculated.

## Results

### Literature Search

**Figure 1:**
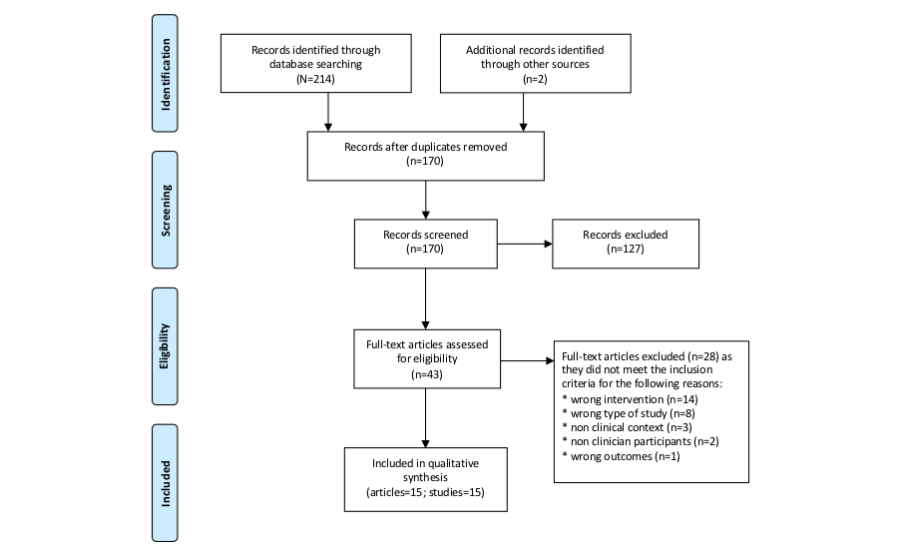
PRISMA (Preferred Reporting Items for Systematic Reviews and Meta-analyses) study flowchart.

A total of 214 citations were retrieved through a structured search of the 5 databases. Two additional records were identified through other sources. After removing duplicates, 170 articles were screened. In total, 2 authors (FE and IS) independently screened the titles and abstracts of 170 records against the inclusion and exclusion criteria, and 43 articles met all criteria. The reviewers were in agreement for 97.6% (166/170) records, and discrepancies were resolved through discussion with a third author (PS). The initial 2 authors (FE and IS) independently reviewed the full text of these articles against the inclusion and exclusion criteria, and 28 articles were excluded. The reviewers were in agreement for 100% (43/43) of the articles. A total of 15 articles met the predefined criteria for inclusion. Note that one article described 2 separate studies [19], and 2 articles described the same study but with a different analytic focus [20,21]; therefore, 15 studies were included in the review. The study flowchart and the reasons for exclusion at each stage are documented in the PRISMA study flowchart in Figure 1.

### Description of Study Characteristics

Table 1 summarizes the 15 included studies. Most studies used a crossover design (10/15, 67%) [19-28], whereas the remainder used a case series design (3/15, 20%) [29-31] or a case study design (2/15, 13%) [32,33]. Approximately half of the studies (8/15, 53%) occurred in a simulated clinical context [19,22-27].

Approximately half of the studies (7/15, 47%) used a volunteer or convenience participant sample [19-21,24,26,29,30], whereas the remainder did not specify how their sample was recruited [22,23,25,27,28,31-33]. The sample size ranged from 2 to 40 participants (median 11.00; mean 12.60, SD 11.35); one study did not report the number of participants (1/15, 7%) [25]. Most of the studies (9/15, 60%) recruited anesthesiologists as participants [19-21,25,26,28-31], and a few studies (3/15, 20%) recruited surgeons [22,24,32]. Most of the studies reported quantitative information about the participants’ medical experience (10/15, 67%) [19,21,23,24,26-28,30,33], and a few studies reported the ages of the participants (3/15, 20%) [24,27,28].

**Table 1:** Summary of the studies included.

Study	Study design	Participants (n); context	Comparison condition	Type of head-worn display	Format of data;source^a^
Beuchat et al, 2005 [22]	Randomized controlled trial with crossover design	Cardiovascular surgeons (n=4); simulated operating room (open heart surgery)	Standard monitoring	Binocular; opacity unspecified; Sony Glasstron	Waveforms and numbers; mirror
Block et al, 1995 [29]	Case series	Anesthesiologists (n=11); operating room (anesthesia)	None	Monocular optical see-through; Reflection Technology Private Eye	Numbers; redesign
Drake-Brockman et al, 2016 [30]	Case series	Anesthesiologists—pediatric (n=40); operating room (anesthesia)	None	Monocular optical see-through; Google Glass	Numbers; redesign
Iqbal et al, 2016 [23]	Crossover trial with fixed order of presentation	Urologists (n=37); simulated operating room (prostatectomy)	Standard monitoring	Monocular optical see-through; Google Glass	Waveforms and numbers; not stated
Liebert et al, 2016 [24]	Randomized controlled trial with crossover design	Surgical residents (n=14); simulated operating room (thoracostomy, bronchoscopy)	Standard monitoring	Monocular optical see-through; Google Glass	Waveforms and numbers; mirror
Liu et al, 2009 [20] and Liu et al, 2010 [21]^b^	Randomized controlled trial with crossover design	Anesthesiologists (n=6); operating room (anesthesia)	Standard monitoring	Monocular optical see-through; Microvision Nomad	Waveforms and numbers; limited replica
Liu et al, 2009 [19]: experiment 1	Crossover design with Latin square assignment to order	Anesthesiologists (n=12); simulated operating room (anesthesia)	Standard monitoring	Monocular optical see-through; Microvision Nomad	Waveforms and numbers; redesign
Liu et al, 2009 [19]: experiment 2	Crossover design with alternating allocation to conditions	Anesthesiologists (n=12); simulated operating room (anesthesia)	Standard monitoring	Monocular optical see-through; Microvision Nomad	Waveforms and numbers; redesign
Ormerod et al, 2002 [25]	Crossover trial with alternative allocation to condition	Anesthesiologists (sample not stated); simulated operating room (anesthesia)	Standard monitoring	Monocular optical see-through; Microvision Nomad	Waveforms and numbers; not stated
Sanderson et al, 2008 [26]	Crossover trial with Latin square assignment to order	Anesthesiologists (n=16); simulated operating room (anesthesia)	Standard monitoring	Monocular optical see-through; Microvision Nomad	Numbers; redesign
Schaer et al, 2015 [27]	Randomized controlled trial with crossover design	Medical residents (n=7); simulated surgical setting (cardiac surgery)	Standard monitoring	Monocular optical see-through; Google Glass	Waveforms and numbers; redesign
Schlosser et al, 2019 [28]	Randomized controlled trial with crossover design	Anesthesiologists—supervising (n=6); operating suite (multiple patient anesthesia)	Standard monitoring	Monocular opaque; Vuzix M300	Waveforms and numbers; redesign
Via et al, 2002 [31]	Case series	Anesthesiologists (n=12); operating room (anesthesia)	None	Binocular optical see-through; Kaiser Electro-Optics	Waveforms and numbers; mirror
Vorraber et al, 2014 [32]	Case study	Surgeons (n=2); operating room (percutaneous transluminal angioplasty)	None	Monocular optical see-through; Google Glass	Waveforms and numbers; mirror
Yoshida et al, 2014 [33]	Case study	Urologists (n=2); operating room (transurethral resection of the prostate)	None	Binocular opaque; Sony HMZ-T2	Waveforms and numbers; redesign

^a^Source refers to whether the *head-worn display* showed vital sign information in the same format as the standard monitor (mirror), a similar format with some vital sign information removed (limited replica), or in a new format (redesign).

^b^Liu et al’s clinical study outcomes were reported over 2 papers; treatment here integrates findings from both studies [20,21].

### Description of HWD Type and Interface

Table 1 summarizes the types of HWD and how the data were displayed. Most of the studies (12/15, 80%) used monocular HWDs [19-21,23-30,32], and the remainder used binocular HWDs (3/15, 20%) [22,31,33]. Of the studies using monocular HWDs, 8% (1/12) used an opaque HWD [28], whereas the remaining studies used optical see-through HWDs (11/12, 92%). Of the 3 studies using binocular HWDs, the HWD in one study (1/3, 33%) was opaque [33], creating an immersive virtual reality experience; in another study, it was optical see-through (1/3, 33%) [31]; and the third study did not specify whether the background was opaque or transparent (1/3, 33%) [22].

Of 15 studies, 12 (75%) displayed a combination of waveforms and numbers on the HWD [19-25,27,28,31-33] and 3 (20%) presented only numbers [26,29,30]. Only 27% (4/15) of the studies presented identical data in the same format on the HWD as on the standard patient monitor [22,24,31,32]. Of 15 studies, 1 (7%) presented on the HWD a subset of the data available on the standard patient monitor [20,21]. Approximately half (8/15, 53%) of the studies presented identical data on the HWD as on the standard monitor but in a redesigned format [19,26-30,33]. Of 15 studies, 2 (13%) studies did not state whether the data were in the same format as the patient monitor or in a redesigned format [23,25], and only 1 (7%) study conducted a formal requirements analysis before implementing and testing the HWD [32].

### Risk of Bias of Included Studies

For the 13 studies with quantitative measures, quality ratings [18] ranged from 25% to 92%, with a median rating of 75%. For the 3 studies with qualitative measures, quality ratings ranged from 30% to 75%, with a median rating of 39%. Detailed quality assessments are included in Table S6 in Multimedia Appendix 1.

### Description of Findings for Clinical Outcomes

Table 2 shows the outcomes extracted from each study. A table with the same data in an extended format is shown in Table S7 in Multimedia Appendix 1. We examined papers for any reports of 3 patient-related clinical outcomes: mortality, morbidity, and rate of complications. Although 7 studies were performed with human patients in clinical contexts rather than simulated patients [21,28-33], none of these studies provided any data relating to the clinical outcomes mentioned earlier.

**Table 2:** Summary of the clinical, surrogate, and process outcomes considered in each study.

Study	Clinical outcomes	Surrogate outcome categories^a^	Process outcome categories^a^	Quality rating (%)	
				Quantitative measure	Qualitative measure
Beuchat et al, 2005 [22]	N/A^b^	+ Decreased missed vital sign changes + Reduced time to detect vital sign changes	+ Changed patterns of gaze behavior + Increased time focused on patient	58	—^c^
Block et al, 1995 [29]	N/M^d^	N/M	+ Clinician opinions	25	—
Drake-Brockman et al, 2016 [30]	N/M	N/M	+ Clinician opinions	83	—
Iqbal et al, 2016 [23]	N/A	+ Reduced time to detect vital sign changes	= Changed time to do other tasks + Clinician opinions	63	—
Liebert et al, 2016 [24]	N/A	= Earlier identification of deterioration	+ Changed patterns of gaze behavior + Increased time focused on patient	92	—
Liu et al, 2009 [20] and Liu et al, 2010 [21]^e^	N/M	N/M	+= Changed patterns of gaze behavior + Increased time focused on patient = Clinician opinions	88	—
Liu et al, 2009 [19]: experiment 1	N/A	= Increased unexpected events detected = Reduced time to detect unexpected events	+ Changed patterns of gaze behavior + Increased time focused on patient = Clinician opinions	75	—
Liu et al, 2009 [19]: experiment 2	N/A	+= Increased unexpected events detected += Reduced time to detect unexpected events	+ Changed patterns of gaze behavior + Increased time focused on patient + Clinician opinions	75	—
Ormerod et al, 2002 [25]	N/A	N/M	+ Changed patterns of gaze behavior + Increased time focused on patient + Changed time to do other tasks + Clinician opinions	29	—
Sanderson et al, 2008 [26]	N/A	= Increased unexpected events detected = Reduced time to detect unexpected events	+ Clinician opinions	83	—
Schaer et al, 2015 [27]	N/A	N/M	+ Clinician opinions	71	—
Schlosser et al, 2019 [28]	N/M	+ Increased alarms detected = Reduced time to detect alarms	+− Clinician opinions	92	75
Via et al, 2002 [31]	N/M	N/M	+ Clinician opinions	59	—
Vorraber et al, 2014 [32]	N/M	N/M	+ Changed patterns of gaze behavior + Clinician opinions	—	30
Yoshida et al, 2014 [33]	N/M	N/M	+ Clinician opinions	—	39

^a^“+” represents the positive effect of head-worn display; “−” represents the negative effect of head-worn display; “=” represents no difference between head-worn display and other conditions. If a study used more than one measure for an outcome, multiple symbols are shown.

^b^N/A: not applicable.

^c^Not relevant to this study.

^d^N/M: not measured.

^e^Liu et al’s clinical study outcomes were reported over 2 papers; treatment here integrates findings from both studies [20,21].

### Description of Findings for Surrogate Outcomes

We extracted data for 9 predetermined surrogate outcomes that were subsequently divided into 4 categories: (1) vital sign changes, (2) alarm detection, (3) unexpected event detection, and (4) situation awareness.

#### Vital Sign Changes

Of 15 studies, 3 (20%) examined the participants’ ability to detect vital sign changes in simulated surgical settings. Overall, 2 of the studies found that the average time to detect abnormal vital signs was significantly faster with an HWD than with standard monitoring. In the first study, Beuchat et al [22] found that participants using an HWD responded almost twice as fast as participants using conventional monitoring, although the overall duration of the surgical intervention was the same for HWD and conventional monitoring. Beuchat et al [22] also found that abnormal vital signs were always detected by participants with an HWD but not by participants using conventional monitoring.

In the second study, Iqbal et al [23] reported that 84% of participants using the HWD responded faster to abnormal vital signs than participants in the conventional monitoring group; however, measures of technical performance from the simulation, including a measure of simulated blood loss, were similar for participants across groups. In the third study, Liebert et al [24] found only a nonsignificant trend for participants using the HWD to recognize abnormal vital signs faster than participants using a standard monitor. Overall, there is some evidence that HWDs can improve participants’ detection of vital sign changes.

#### Alarms

One study examined whether the use of an HWD increased the number of auditory alarms detected by clinicians or reduced the time taken for clinicians to detect alarms. Schlosser et al [28] delivered alarms in visual and auditory formats via the HWD and found that anesthesiologists supervising work in 6 operating rooms noticed a higher percentage of alarms when using the HWD than when relying on central monitoring or on monitoring within each operating room (67% compared with 7%). There was no difference between the conditions in the median time taken to detect alarms.

#### Unexpected Events

Of 15 studies, 3 (20%) examined the effect of HWDs on the detection of unexpected events and the time taken to respond to these events. For unexpected events occurring on the HWD, such as hypertension or gas embolism, 2 simulator studies found that participants using the HWD did not detect more unexpected events [26] and did not detect them faster than participants using standard monitoring [19,26]. In a third simulator study, Liu et al [19] (experiment 2), anesthesiologists worked under conditions where they were either operationally constrained (by being involved in an intubation task) or physically constrained (by requiring 2 hands to operate a surgical tool). The HWD increased the detection rate and speed of detection in some clinical scenarios (tachypnea or hypertension and hypotension events) but not others (hypoventilation), compared with standard monitoring.

Liu et al [19] also examined unexpected events occurring outside the HWD, such as the simulated patient opening their eyes while anesthetized or a medical student fainting. Participants using the HWD did not detect events faster than the participants using standard monitoring. Overall, the HWD does not appear to improve participants’ ability to notice unexpected events unless participants are constrained from noticing them on a standard monitor.

#### Situation Awareness

Our intended criterion for including situation awareness outcomes was that a validated measure should be used; none of the studies met this criterion. However, one study described nonvalidated measures of situation awareness, specifically self-reported awareness, and are reported here for completeness. Schlosser et al [28] surveyed clinicians after they used an HWD and found that they reported that the HWD helped them to comprehend the environment and make assessments more easily and that information gained from the HWD affected future actions.

### Description of Findings for Process Outcomes

We extracted data for 5 predetermined process outcomes that were divided into 4 categories: (1) gaze behavior, (2) time for other tasks, (3) information sharing, and (4) clinicians’ opinions of HWDs.

#### Gaze Behavior

Of 15 studies, 7 (47%) examined the gaze behavior of clinicians using an HWD. Of 7 studies, 5 found that clinicians using an HWD spent more time looking at the patient or the procedural field and less time looking at the monitor than when the HWD was not used [19,21,24,25,32] and 2 found that clinicians using an HWD showed a decrease in head movements or shifts in attention, compared with those using standard monitoring [22,25]. A further analysis of the data in a study by Liu et al [21] indicated that when using an HWD, clinicians spent more time looking toward the patient and less time looking toward an anesthesia machine than when they used standard monitoring for some clinical scenarios (eg, anesthesia crisis management) but not for other scenarios (anesthesia confirmation of laryngeal mask airway placement) [20].

#### Time Spent on Tasks

Overall, 2 studies examined the effect of using an HWD on the time spent on tasks. In one study, Ormerod et al [25] found that clinicians using an HWD spent less time completing anesthesiology tasks than clinicians using standard monitoring, with the implication that the HWD removed the need for clinicians to interrupt tasks to view a monitor. In another study, Iqbal et al [23] found that clinicians using an HWD performed a surgical task at a similar speed to clinicians using a standard monitor.

#### Information Sharing

None of the studies considered the effect of HWDs on how clinicians share information.

#### Clinicians’ Opinions

Of 15 studies, 14 (93%) examined some aspects of clinicians’ opinions of HWDs for vital sign monitoring [19,21,23-33]. Overall, clinicians had positive opinions about the use of HWDs. In 10 studies, clinicians were asked about the ease of reading or interpretation; in 7 (70%) of those studies, most clinicians reported that the HWD was easy to read or interpret [19,24-28,30]. However, one study found no difference in ease of monitoring between clinicians using HWDs and clinicians using standard monitoring [21], and one study found that approximately half of the clinicians preferred an HWD whereas half preferred using the standard monitor [19].

In 6 studies, clinicians were asked whether wearing the HWD was comfortable. In 5 of these studies, most clinicians reported that the HWD was comfortable to wear or did not report any discomfort [23,29,30,32,33]. However, in one study, half of the clinicians reported that the HWD was too big or too heavy, and they experienced discomfort or pain from wearing it [28].

Finally, in all 6 studies where clinicians were asked whether they would use the device again, most clinicians said yes, although sometimes noting improvements needed to the device or to the information presented [23,24,28-31].

## Discussion

### Principal Findings

The purpose of this review was to evaluate the impact of HWDs displaying continuous vital sign monitoring on clinical, surrogate, and process outcomes in critical and acute care contexts. Our systematic review of the literature shows that HWDs have been evaluated for continuous vital sign monitoring in 15 studies, including 7 conducted with patients in clinical environments. Clearly, HWDs can be technically implemented for vital sign monitoring, but the evidence for any overall benefit is mixed. None of the 7 clinical studies measured clinical outcomes. Across all 15 studies, there was only limited evidence that HWDs displaying patient vital signs improve surrogate outcomes or process outcomes.

The strongest and most consistent evidence for the benefit of HWDs relates to gaze behavior. Several studies have shown that wearing an HWD tends to increase the time that clinicians spend looking toward the patient relative to the patient monitor or anesthesia machine [19-22,24,25,32]. However, as Liu et al [19] pointed out, when the HWD image overlays the view of a patient, a clinician may be looking toward a patient but directing their attention to the HWD image rather than to the patient. Therefore, although the evidence for changes in gaze behavior toward the patient is consistent, it does not necessarily imply greater attention to the patient.

There is also consistent evidence that clinicians using an HWD may take less time to detect abnormal vital signs and may also detect more vital sign changes than clinicians using standard monitoring. However, this evidence comes from a limited number of studies—2 with significant results [22,23] and one with a nonsignificant trend [24]—and is therefore not conclusive.

Evidence for whether HWDs affect clinicians’ detection of unexpected events signaled on an HWD is mixed. In 2 studies, there was no difference between HWDs and standard monitoring for detecting unexpected events [19,26]. Liu et al [19] reported that in operationally constrained conditions, HWDs may increase the detection rate and speed of detection of unexpected events compared with standard monitoring. Specifically, when vital signs indicating an unexpected event were presented as numbers, the HWD increased the detection of unexpected events. However, when the relevant vital sign was presented as a waveform, the HWD worsened detection. This suggests that when vital sign information on the HWD is in the forward field of view, certain formats may actually be less detectible compared with the same information presented on a standard monitor. Liu et al [19] found no difference between HWDs and standard monitoring in how often clinicians noticed unexpected events occurring in the simulated operating room rather than on the HWD.

For other outcomes, the evidence is mixed. HWDs are sometimes associated with improvements in alarm detection and the time required to complete tasks, but sometimes not. This makes it difficult to provide definitive conclusions regarding the effects that HWDs have on these outcomes. Further research is needed to determine the factors that might moderate the effect of HWDs on these outcomes. For situation awareness, the fact that no study has collected objective measures of the 3 levels of situation awareness by Endsley [34,35]—how HWDs affect clinicians’ perception and comprehension of the patient’s current state and their projection of the patient’s future state—means we do not yet know the impact of HWDs on situation awareness. Subjective and observational evidence suggests that HWDs may improve clinicians’ awareness of the ongoing situation [28], but further research is needed to verify this claim.

### Future Research

For more definitive conclusions to be drawn about the impact of HWDs on vital sign monitoring, the focus and quality of the research need to be improved. The quality assessment ratings ranged from 25% to 92%, indicating that there is room for improvement in study design and methods. Given that there is no strong evidence that HWDs worsened performance on any outcome measures reported, it is worth investing in higher-fidelity studies to improve the evidence base.

The inconsistent results may be partly explained by the use of small-scale, short-term studies with a focus on surrogate and process outcomes. Longitudinal studies may allow clinicians to adjust to the novelty of using an HWD and may reveal whether the benefits of HWDs emerge over time. Large-scale randomized control trials have not yet emerged, but clinically based studies focusing on clinically relevant outcomes will help clinical leaders decide whether HWDs should be implemented on a large-scale basis.

In the literature reviewed, there was little apparent attention to user-centered design principles [36] when researchers designed and tested the HWD display format and content for critical and acute care environments. A user-centered design approach would help developers to focus on the outcomes that the HWD might improve. The implementation of user-centered design principles would involve determining the requirements that the system (ie, the HWD) must meet to achieve the specified outcomes [37], designing the system to meet those requirements [38], and then conducting empirical tests with users to determine if the requirements have indeed been met and outcomes, achieved. The process is repeated until the requirements are met. Given that users’ opinions about a device or technology can sometimes differ markedly from their performance when using that device or technology [39-41] and that it is the performance that will address the outcomes, it is essential to perform robust empirical tests.

No study in this literature review reported a systematic analysis of user needs. A requirements analysis was reported in only one of the 15 studies (Vorraber et al [32]) before designing and testing the HWD system. The largely positive feedback from clinicians reported in the studies reviewed may indicate that they discern uses and usefulness of HWDs that have not yet materialized in practice or they may be overlooking requirements that, without a prospective requirements analysis, would only emerge if HWDs were fully integrated into practice.

An examination of the HWD interfaces used in the literature also supports the assertion that a user-centered design approach may be worthwhile in future research. Of the 13 studies that reported the layout of the HWD interface, 4 presented the data in the same format on the HWD as on the standard patient monitor [22,24,31,32] and one provided a limited replica [20,21]. The remaining 8 studies redesigned the format of the information [19,26-30,33]. Interestingly, the study by Vorraber et al [32], which included a requirements analysis, used the same format as the standard patient monitor. However, this does not indicate that the same format is appropriate for every clinical context. In studies that used a redesigned format, clinicians tended to prefer an HWD display layout in the same format as the standard monitor, but this was not always the case. In one study, most clinicians reported that they would use the HWD again but that they wanted more information on the display [30]. In another study, 82% of clinicians said they would use the HWD again if it displayed waveforms and numbers in the same format as the standard monitor [29]. However, in a further study, clinicians reported that they found information on the HWD redundant with the information on the standard monitor [26]. It may be that each of these studies represents a different use case. If so, performing a requirements analysis motivated by desired outcomes would guide the design of HWD software and hardware for specific clinical contexts. When coupled with longitudinal evaluation studies, this approach could lead to clearer and more consistent results.

Evaluations of the impact of HWDs would be improved if researchers include tests that allow evidence to accumulate for or against the key outcomes listed in this review. For example, relatively few studies have tested whether HWDs render participants more or less able to detect changes in patient vital signs or to notice unexpected events. If future studies were to deliberately augment evidence for or against key claims, clinical leaders would be able to make more confident decisions about the viability of HWDs.

### Strengths and Limitations of the Review

The purpose of this systematic review was to evaluate the benefits or otherwise of HWDs for continuous vital sign monitoring, using a set of predefined outcome categories. The strengths of the review are its scope and the breadth of outcomes considered for each study, revealing the considerable heterogeneity of approaches and findings in the area, and indicating areas for further research.

This review has several limitations. First, the heterogeneous nature of the literature poses a challenge to interpretation. Our search terms may not have captured all relevant publications if different key terms are used across different clinical contexts. However, we tried to address this through an iterative process for developing search terms in collaboration with librarians and clinicians. Second, the quality of evidence ranged considerably across the included studies. Third, there may be evidence in other contexts that HWDs can improve or worsen performance that is relevant to continuous vital sign monitoring in acute and critical care contexts; however, finding such cases was outside the scope of the review. Finally, there are other ways to convey continuously captured vital signs to clinicians. For example, auditory, haptic, or multimodal displays may meet clinicians’ requirements for continuous patient monitoring. However, this was beyond the scope of this review.

### Conclusions

Certain surrogate and process outcomes suggest that HWDs may assist continuous vital sign monitoring, but to date, there have been no evaluations of whether HWDs improve clinical outcomes. The most consistent evidence across the corpus of studies reviewed is that HWDs can improve clinicians’ detection of vital sign changes and reduce the time clinicians spend looking at the patient monitor. However, for other surrogate and process outcomes, the evidence is mixed. A user-centered design approach can produce designs and evaluations that are more focused on the desired outcomes. Further research is required to determine whether, in what contexts, and under what conditions HWDs can reliably support the early recognition of patient deterioration and potentially reduce patient harm.

## Appendix

Multimedia Appendix 1 Supplementary material including search terms, search strategy, a full list of outcome questions, study inclusion and exclusion criteria, data extraction categories, the full quality assessment for all included studies, and a summary table of the clinical, surrogate, and process outcomes assessed in each study.

## Abbreviations

HWD: head-worn display
PRISMA: Preferred Reporting Items for Systematic Reviews and Meta-analyses
