# Supplementary material for: The Use of Head-Worn Displays for Vital Sign Monitoring in Critical and Acute Care: Systematic Review
Source: JMIR Mhealth Uhealth. 2021 May 11;9(5):e27165. doi: 10.2196/27165 (PMC8150412; doi:10.2196/27165)
Supplement: Multimedia Appendix 1 [file mhealth_v9i5e27165_app1.docx]

**Supplementary material**

# Table S1: Search terms

| **Concept** | **Search term** |
| --- | --- |
| Vital sign monitoring | ("vital signs" OR "vital sign" OR vitals OR monitoring OR ECG OR electrocardiogra* OR heart OR "blood pressure" OR capnogra* OR “end-tidal” OR “end tidal” OR pulse OR respirat* OR oximet* OR saturation OR temperature) |
|  | AND |
| Head worn displays | ("head worn display" OR “headworn display” OR “head-worn display” OR HWD OR "head mounted display" OR “headmounted display” OR “head-mounted display” OR (HMD NOT Hyaline membrane disease) OR "head up display" OR “headup display” OR “head-up display” OR “heads up display” OR “headsup display” OR “heads-up display” OR "wearable headset" OR "smart glasses" OR smartglasses OR "google glass" OR vuzix OR Fountx) |
|  | AND |
| Patients | patient |

# Table S2: Search strategy (full record of search)

## PubMed

Search date: 13/06/2019, 15/08/2020

Search strategy:

| **Step** | **Query** | **Results** | **Input** |
| --- | --- | --- | --- |
| 1 | "vital signs"[MeSH Terms] OR "vital sign" OR vitals OR monitoring OR ECG OR electrocardiogra* OR heart OR "blood pressure" OR capnogra* OR “end-tidal” OR “end tidal” OR pulse OR respirat* OR oximet* OR saturation OR patient OR temperature | 3832816 | Vital sign search term |
| 2 | "head worn display" OR “headworn display” OR “head-worn display” OR HWD OR "head mounted display" OR “headmounted display” OR “head-mounted display” OR (HMD NOT Hyaline membrane disease) OR "head up display" OR “headup display” OR “head-up display” OR “heads up display” OR “headsup display” OR “heads-up display” OR "wearable headset" OR "smart glasses" OR smartglasses OR "google glass" OR vuzix OR Fountx | 1396 | Head worn displays search term |
| 3 | patient | 6580918 | Patient search term |
| 4 | (((("vital signs"[MeSH Terms] OR "vital sign" OR vitals OR monitoring OR ECG OR electrocardiogra* OR heart OR "blood pressure" OR capnogra* OR “end-tidal” OR “end tidal” OR pulse OR respirat* OR oximet* OR saturation OR temperature))) AND (("head worn display" OR “headworn display” OR “head-worn display” OR HWD OR "head mounted display" OR “headmounted display” OR “head-mounted display” OR (HMD NOT Hyaline membrane disease) OR "head up display" OR “headup display” OR “head-up display” OR “heads up display” OR “headsup display” OR “heads-up display” OR "wearable headset" OR "smart glasses" OR smartglasses OR "google glass" OR vuzix OR Fountx))) AND patient | 65 | Combined search term |
| 5 | (((("vital signs"[MeSH Terms] OR "vital sign" OR vitals OR monitoring OR ECG OR electrocardiogra* OR heart OR "blood pressure" OR capnogra* OR “end-tidal” OR “end tidal” OR pulse OR respirat* OR oximet* OR saturation OR temperature))) AND (("head worn display" OR “headworn display” OR “head-worn display” OR HWD OR "head mounted display" OR “headmounted display” OR “head-mounted display” OR (HMD NOT Hyaline membrane disease) OR "head up display" OR “headup display” OR “head-up display” OR “heads up display” OR “headsup display” OR “heads-up display” OR "wearable headset" OR "smart glasses" OR smartglasses OR "google glass" OR vuzix OR Fountx))) AND patient Filters: Publication date from 1995/01/01 to 2020/06/30 | 60 | Add filter for publication dates 1995 - 2020 |
| 6 | (((("vital signs"[MeSH Terms] OR "vital sign" OR vitals OR monitoring OR ECG OR electrocardiogra* OR heart OR "blood pressure" OR capnogra* OR “end-tidal” OR “end tidal” OR pulse OR respirat* OR oximet* OR saturation OR temperature))) AND (("head worn display" OR “headworn display” OR “head-worn display” OR HWD OR "head mounted display" OR “headmounted display” OR “head-mounted display” OR (HMD NOT Hyaline membrane disease) OR "head up display" OR “headup display” OR “head-up display” OR “heads up display” OR “headsup display” OR “heads-up display” OR "wearable headset" OR "smart glasses" OR smartglasses OR "google glass" OR vuzix OR Fountx))) AND patient Filters: Publication date from 1995/01/01 to 2020/06/30; Other Animals | 2 | Add filter for “other animals” (i.e., non human animals) |
| 7 | ((((((("vital signs"[MeSH Terms] OR "vital sign" OR vitals OR monitoring OR ECG OR electrocardiogra* OR heart OR "blood pressure" OR capnogra* OR “end-tidal” OR “end tidal” OR pulse OR respirat* OR oximet* OR saturation OR temperature))) AND (("head worn display" OR “headworn display” OR “head-worn display” OR HWD OR "head mounted display" OR “headmounted display” OR “head-mounted display” OR (HMD NOT Hyaline membrane disease) OR "head up display" OR “headup display” OR “head-up display” OR “heads up display” OR “headsup display” OR “heads-up display” OR "wearable headset" OR "smart glasses" OR smartglasses OR "google glass" OR vuzix OR Fountx))) AND patient) AND ( "1995/01/01"[PDat] : "2019/12/31"[PDat] ))) NOT (((((("vital signs"[MeSH Terms] OR "vital sign" OR vitals OR monitoring OR ECG OR electrocardiogra* OR heart OR "blood pressure" OR capnogra* OR “end-tidal” OR “end tidal” OR pulse OR respirat* OR oximet* OR saturation OR temperature))) AND (("head worn display" OR “headworn display” OR “head-worn display” OR HWD OR "head mounted display" OR “headmounted display” OR “head-mounted display” OR (HMD NOT Hyaline membrane disease) OR "head up display" OR “headup display” OR “head-up display” OR “heads up display” OR “headsup display” OR “heads-up display” OR "wearable headset" OR "smart glasses" OR smartglasses OR "google glass" OR vuzix OR Fountx))) AND patient) AND ( "1995/01/01"[PDat] : "2020/06/30"[PDat] ) AND Animals[Mesh:noexp]) | 58 | Remove articles via filter for “other animals” using the NOT function. |
| 8 | ((((((("vital signs"[MeSH Terms] OR "vital sign" OR vitals OR monitoring OR ECG OR electrocardiogra* OR heart OR "blood pressure" OR capnogra* OR “end-tidal” OR “end tidal” OR pulse OR respirat* OR oximet* OR saturation OR temperature))) AND (("head worn display" OR “headworn display” OR “head-worn display” OR HWD OR "head mounted display" OR “headmounted display” OR “head-mounted display” OR (HMD NOT Hyaline membrane disease) OR "head up display" OR “headup display” OR “head-up display” OR “heads up display” OR “headsup display” OR “heads-up display” OR "wearable headset" OR "smart glasses" OR smartglasses OR "google glass" OR vuzix OR Fountx))) AND patient) AND ( "1995/01/01"[PDat] : "2020/06/30"[PDat] ))) NOT (((((("vital signs"[MeSH Terms] OR "vital sign" OR vitals OR monitoring OR ECG OR electrocardiogra* OR heart OR "blood pressure" OR capnogra* OR “end-tidal” OR “end tidal” OR pulse OR respirat* OR oximet* OR saturation OR temperature))) AND (("head worn display" OR “headworn display” OR “head-worn display” OR HWD OR "head mounted display" OR “headmounted display” OR “head-mounted display” OR (HMD NOT Hyaline membrane disease) OR "head up display" OR “headup display” OR “head-up display” OR “heads up display” OR “headsup display” OR “heads-up display” OR "wearable headset" OR "smart glasses" OR smartglasses OR "google glass" OR vuzix OR Fountx))) AND patient) AND ( "1995/01/01"[PDat] : "2019/12/31"[PDat] ) AND Animals[Mesh:noexp]) Filters: Publication date from 1995/01/01 to 2020/06/30; English | **57** | Add filter for English language |

## Embase

Search date: 13/06/2019, 15/08/2020

Search strategy:

| **Step** | **Query** | **Results** | **Input** |
| --- | --- | --- | --- |
| 1 | 'vital sign'/exp OR 'vital sign' OR vitals OR 'monitoring'/exp OR monitoring OR 'ecg'/exp OR ecg OR electrocardiogra* OR 'heart'/exp OR heart OR 'blood pressure'/exp OR 'blood pressure' OR capnogra* OR 'end-tidal' OR 'end tidal' OR 'pulse'/exp OR pulse OR respirat* OR oximet* OR 'saturation'/exp OR saturation OR 'temperature'/exp OR temperature | 5,672,350 | Vital sign monitoring concept |
| 2 | 'head worn display' OR 'headworn display' OR 'head-worn display' OR hwd OR 'head mounted display'/exp OR 'head mounted display' OR 'headmounted display' OR 'head-mounted display' OR (('hmd'/exp OR hmd) NOT ('hyaline'/exp OR hyaline) AND ('membrane'/exp OR membrane) AND ('disease'/exp OR disease)) OR 'head up display' OR 'headup display' OR 'head-up display' OR 'heads up display' OR 'headsup display' OR 'heads-up display' OR 'wearable headset' OR 'smart glasses' OR smartglasses OR 'google glass'/exp OR 'google glass' OR vuzix OR fountx | 1,331 | Head worn displays concept |
| 3 | patient | 5,428,763 | Patient concept |
| 4 | #1 AND #2 AND #3 | 80 | Combined search term |
| 5 | #4 AND (1995:py OR 1999:py OR 2000:py OR 2002:py OR 2003:py OR 2004:py OR 2005:py OR 2008:py OR 2009:py OR 2010:py OR 2011:py OR 2012:py OR 2013:py OR 2014:py OR 2015:py OR 2016:py OR 2017:py OR 2018:py OR 2019:py OR 2020:py) | 80 | Add filter for publication dates 1995 - 2020 |
| 6 | #4 AND (1995:py OR 1999:py OR 2000:py OR 2002:py OR 2003:py OR 2004:py OR 2005:py OR 2008:py OR 2009:py OR 2010:py OR 2011:py OR 2012:py OR 2013:py OR 2014:py OR 2015:py OR 2016:py OR 2017:py OR 2018:py OR 2019:py OR 2020:py) AND ([animal cell]/lim OR [animal experiment]/lim OR [animal model]/lim OR [animal tissue]/lim) | 0 | Select all non-human articles |
| 7 | #5 NOT #6 | 80 | Exclude all non-human articles using the NOT function. |
| 8 | #5 NOT #6 AND [english]/lim | 79 | Add filter for English language |
| 9 | #8 AND [embase]/lim NOT ([embase]/lim AND [medline]/lim) | **37** | Exclude articles from medline (included in PubMed) |

## CINHAHL via EBSCOhost

Search date: 13/06/2019, 15/08/2020

Search strategy:

| **Step** | **Query** | **Results** | **Input** |
| --- | --- | --- | --- |
| 1 | MH "Vital Signs+" OR "vital sign" OR vitals OR monitoring OR ECG OR electrocardiogra* OR heart OR "blood pressure" OR capnogra* OR “end-tidal” OR “end tidal” OR pulse OR respirat* OR oximet* OR saturation OR temperature | 660,176 | Vital sign monitoring concept |
| 2 | "head worn display" OR “headworn display” OR “head-worn display” OR HWD OR "head mounted display" OR “headmounted display” OR “head-mounted display” OR (HMD NOT Hyaline membrane disease) OR "head up display" OR “headup display” OR “head-up display” OR “heads up display” OR “headsup display” OR “heads-up display” OR "wearable headset" OR "smart glasses" OR smartglasses OR "google glass" OR vuzix OR Fountx | 411 | Head worn displays concept |
| 3 | patient | 1,770,646 | Patient search term |
| 4 | S1 AND S2 AND S3 | 32 | Combined search term |
| 5 | S1 AND S2 AND S3  Limiters - Published Date: 19950101-20200630 | 31 | Add filter for publication dates 1995 - 2019 |
| 6 | No filter for animal studies | 31 | - |
| 7 | No filter for animal studies | 31 | - |
| 8 | S1 AND S2 AND S3  Limiters - Published Date: 19950101-20200630; English Language | 31 | Add filter for English language |
| 9 | S1 AND S2 AND S3  Limiters - Published Date: 19950101-20200630; English Language; Exclude MEDLINE records | **15** | Exclude Medline records (included in PubMed) |

## PsychINFO

Search date: 13/06/2019, 15/08/2020

Search strategy:

| **Step** | **Query** | **Results** | **Input** |
| --- | --- | --- | --- |
| 1 | Any Field: "vital sign" OR vitals OR monitoring OR ECG OR electrocardiogra* OR heart OR "blood pressure" OR capnogra* OR “end-tidal” OR “end tidal” OR pulse OR respirat* OR oximet* OR saturation OR temperature | 202,114 | Vital sign concept |
| 2 | Any Field: "head worn display" OR “headworn display” OR “head-worn display” OR HWD OR "head mounted display" OR “headmounted display” OR “head-mounted display” OR (HMD NOT Hyaline membrane disease) OR "head up display" OR “headup display” OR “head-up display” OR “heads up display” OR “headsup display” OR “heads-up display” OR "wearable headset" OR "smart glasses" OR smartglasses OR "google glass" OR vuzix OR Fountx | 416 | Head worn displays concept |
| 3 | patient | 332,032 | Patient concept |
| 4 | Any Field: "vital sign" OR vitals OR monitoring OR ECG OR electrocardiogra* OR heart OR "blood pressure" OR capnogra* OR “end-tidal” OR “end tidal” OR pulse OR respirat* OR oximet* OR saturation OR temperature AND Any Field: "head worn display" OR “headworn display” OR “head-worn display” OR HWD OR "head mounted display" OR “headmounted display” OR “head-mounted display” OR (HMD NOT Hyaline membrane disease) OR "head up display" OR “headup display” OR “head-up display” OR “heads up display” OR “headsup display” OR “heads-up display” OR "wearable headset" OR "smart glasses" OR smartglasses OR "google glass" OR vuzix OR Fountx AND Any Field: patient | 6 | Combined search term |
| 5 | Any Field: "vital sign" OR vitals OR monitoring OR ECG OR electrocardiogra* OR heart OR "blood pressure" OR capnogra* OR “end-tidal” OR “end tidal” OR pulse OR respirat* OR oximet* OR saturation OR temperature AND Any Field: "head worn display" OR “headworn display” OR “head-worn display” OR HWD OR "head mounted display" OR “headmounted display” OR “head-mounted display” OR (HMD NOT Hyaline membrane disease) OR "head up display" OR “headup display” OR “head-up display” OR “heads up display” OR “headsup display” OR “heads-up display” OR "wearable headset" OR "smart glasses" OR smartglasses OR "google glass" OR vuzix OR Fountx AND Any Field: patient AND Year: 1995 To 2020 | 6 | Add filter for publication dates from 1995 to 2019 |
| 6 | Any Field: "vital sign" OR vitals OR monitoring OR ECG OR electrocardiogra* OR heart OR "blood pressure" OR capnogra* OR “end-tidal” OR “end tidal” OR pulse OR respirat* OR oximet* OR saturation OR patient OR temperature AND Any Field: "head worn display" OR “headworn display” OR “head-worn display” OR HWD OR "head mounted display" OR “headmounted display” OR “head-mounted display” OR (HMD NOT Hyaline membrane disease) OR "head up display" OR “headup display” OR “head-up display” OR “heads up display” OR “headsup display” OR “heads-up display” OR "wearable headset" OR "smart glasses" OR smartglasses OR "google glass" OR vuzix OR Fountx AND Population Group: Animal AND Year: 1995 To 2020 | 0 | Add filter for animal studies - unsuccessful |
| 7 | Excluding by filtering for animal studies unsuccessful | 6 | - |
| 8 | Any Field: "vital sign" OR vitals OR monitoring OR ECG OR electrocardiogra* OR heart OR "blood pressure" OR capnogra* OR “end-tidal” OR “end tidal” OR pulse OR respirat* OR oximet* OR saturation OR temperature AND Any Field: "head worn display" OR “headworn display” OR “head-worn display” OR HWD OR "head mounted display" OR “headmounted display” OR “head-mounted display” OR (HMD NOT Hyaline membrane disease) OR "head up display" OR “headup display” OR “head-up display” OR “heads up display” OR “headsup display” OR “heads-up display” OR "wearable headset" OR "smart glasses" OR smartglasses OR "google glass" OR vuzix OR Fountx AND Any Field: patient AND Language: English AND Year: 1995 To 2020 | 6 | Add filter (via field search) for English language |
| 9 | No filter for source | **6** | - |

## Web of Science

Search date: 13/06/2019, 15/08/2020

Search strategy:

| **Step** | **Search** | **No. results** | **Input** |
| --- | --- | --- | --- |
| 1 | ALL=("vital sign" OR vitals OR monitoring OR ECG OR electrocardiogra* OR heart OR "blood pressure" OR capnogra* OR "end-tidal" OR "end tidal" OR pulse OR respirat* OR oximet* OR saturation OR temperature) | 7,859,852 | Vital sign concept |
| 2 | ALL=("head worn display" OR “headworn display” OR “head-worn display” OR HWD OR "head mounted display" OR “headmounted display” OR “head-mounted display” OR (HMD NOT Hyaline membrane disease) OR "head up display" OR “headup display” OR “head-up display” OR “heads up display” OR “headsup display” OR “heads-up display” OR "wearable headset" OR "smart glasses" OR smartglasses OR "google glass" OR vuzix OR Fountx) | 5,352 | Head worn display concept |
| 3 | ALL=(patient) | 5,959,608 | Patient concept |
| 4 | #3 AND #2 AND #1 | 106 | Combined search tem |
| 5 | #3 AND #2 AND #1  Indexes=SCI-EXPANDED, SSCI, A&HCI, CPCI-S, CPCI-SSH, BKCI-S, BKCI-SSH, ESCI, CCR-EXPANDED, IC Timespan=1995-2020 | 102 | Add filter for publication dates from 1995 to 2020 |
| 6 | No filter for animal studies | 102 | - |
| 7 | No filter for animal studies | 102 | - |
| 8 | (#3 AND #2 AND #1) AND LANGUAGE: (English)  Indexes=SCI-EXPANDED, SSCI, A&HCI, CPCI-S, CPCI-SSH, BKCI-S, BKCI-SSH, ESCI, CCR-EXPANDED, IC Timespan=1995-2020 | 99 | Add filter for English language |
| 9 | No filter for source | **99** | - |

# Table S3: Outcome questions

| **Clinical** | Do HWDs reduce patient mortality? |
| --- | --- |
|  | Do HWDs reduce patient morbidity? |
|  | Do HWDs result in fewer complications? |
| **Surrogate** | Does the use of HWDs assist in the early identification of patient deterioration? |
|  | Does the use of HWDs reduce the number or duration of adverse events experienced by patients? |
|  | Does the use of HWDs decrease the number of vital sign changes missed by clinicians? |
|  | Does the use of HWDs reduce the time taken to detect or respond to vital sign changes? |
|  | Does the use of HWDs increase the number of alarms detected by clinicians? |
|  | Does the use of HWDs reduce the time taken to detect or respond to alarms? |
|  | Does the use of HWDs increase the proportion of unexpected events detected? |
|  | Does the use of HWDs reduce the time taken to detect or respond to unexpected events? |
|  | Does the use of HWDs improve situation awareness (as measured by a validated scale of situation awareness)? |
| **Process** | Does the use of HWDs change clinicians' patterns of gaze behaviour? |
|  | Does the use of HWDs increase time focused on the patient? |
|  | Does the use of HWDs affect the time to do other tasks? |
|  | Does the use of HWDs improve information sharing for clinicians who may be working at a distance from each other? |
|  | What are clinicians' opinions of HWDs for vital sign monitoring? |

# Table S4: Study inclusion and exclusion criteria

|  | **INCLUSION** | **EXCLUSION** |
| --- | --- | --- |
| **Year** | - Published between 1995 and 2020 |  |
| **Language** | - Published in English, or - English abstract |  |
| **Type of study** | - Must be a peer reviewed study, either a:   - Randomised controlled trial   - Controlled trial without randomisation   - Case-control   - Cohort study   - Qualitative or descriptive study   - Mixed method study   - Medical letter-to-the-editor (containing study/ies which fit under the above descriptions)   - Conference abstract, or   - Conference paper | - Grey literature - Reviews - Editorials - Systematic reviews - Meta-analyses - Non-peer reviewed articles - Others that do not fit the inclusion criteria |
| **Context** | - Any critical care or acute care clinical OR simulated clinical context, including:   - pre- hospital retrieval   - emergency department   - intensive care   - peri-operative   - intra-hospital transport   - labour and delivery - Must have continuous patient vital sign monitoring | - Any non-clinical or simulated non-clinical context (e.g., lab studies) - Any non-critical care or non-acute care contexts, such as:   - general wards   - geriatric wards   - mental health units |
| **Participants** | - Qualified clinicians   - nurses   - paramedics   - doctors   - anaesthetic technicians   - respiratory therapists   - cardiopulmonary perfusionists   - physiotherapists   - trainees of these disciplines | - Non-clinicians - Students who have not yet achieved a minimum qualification |
| **Intervention** | - Any head-worn display used for patients' vital sign monitoring   - Transparent/opaque   - Monocular/binocular - Vital signs measured by an electronic monitoring device and re-presented on the HWD, including, but not limited to:   - Heart rate   - Blood pressure   - Waveform data (e.g., ECG and capnographs)   - Alarms generated by monitoring devices | - The transmission and display of images or videos or information *without* vital sign information |
| **Comparator** | - Any   - Portable technology (e.g., tablets/auditory displays)   - Standard monitoring equipment   - No comparator |  |
| **Outcomes** | - Clinical   - Patient mortality   - Patient morbidity   - Complications - Surrogate   - Early detection of patient deterioration   - Number or duration of adverse events   - Number of vital sign changes missed   - Time taken to detect or respond to vital sign changes   - Number of alarms detected   - Time taken to detect or respond to alarms   - Proportion of unexpected events detected   - Time taken to detect or respond to unexpected events   - Situation awareness (using a validated scale) - Process   - Patterns of gaze behaviour   - Time focused on patient   - Time to do other tasks   - Information sharing for clinicians working at a distance   - Clinician opinions |  |

# Table S5: Data extraction categories

| Publication details | Author’s names |
| --- | --- |
|  | Publication year |
|  | Title |
|  | Publication type |
|  | Journal |
|  | DOI |
|  | Study funding source |
|  | Possible conflicts of interest |
| Study methodology | Aim of study |
|  | Study design as described |
|  | Study design classification |
|  | Sampling/recruitment technique |
|  | Total number of participants |
|  | Type of participants |
|  | Age group/clinical experience |
|  | Number of trials per participant/length of exposure |
|  | Setting/context |
|  | Experimental condition |
|  | Comparison condition |
|  | Outcome measures |
|  | Formal needs analysis before testing |
| HWD details | Type of HWD |
|  | Vital sign data |
|  | Type of interface (i.e., a mirror which mimics the existing standard display, a new form of the display; if new – deliberate redesign or replica) |
|  | Format of vital sign data |
|  | Manufacturer and model |
| Study outcomes | Clinical outcomes listed by question |
|  | Surrogate outcomes listed by question |
|  | Process outcomes listed by question |
|  | Strengths reported by authors |
|  | Limitations reported by authors |
|  | Additional strengths |
|  | Additional limitations |

# Table S6: Quality assessments of included studies

## Qualitative Studies

| **Study** | **1. Question or objective sufficiently described?** | **2. Study design evident and appropriate?** | **3. Context for the study clear?** | **4. Connection to a theoretical framework or wider body of knowledge?** | **5. Sampling strategy described, relevant, and justified?** | **6. Data collection methods clearly described and systematic?** | **7. Data analysis clearly described and systematic?** | **8. Use of verification procedure(s) to establish credibility?** | **9. Conclusions supported by the results?** | **10. Reflexivity of the account?** | **Total** | **Summary score (%)** |
| --- | --- | --- | --- | --- | --- | --- | --- | --- | --- | --- | --- | --- |
| Schlosser et al., 2019 | YES | YES | YES | YES | PARTIAL | YES | YES | NO | YES | NO | **15/20** | **75%** |
| Vorraber et al., 2014 | YES | PARTIAL | YES | NO | NO | NO | NO | NO | PARTIAL | NO | **6/20** | **30%** |
| Yoshida et al., 2014 | PARTIAL | YES | YES | NO | NO | NO | N/A | NO | YES | NO | **7/18** | **39%** |

## Quantitative Studies

| **Study** | **1.**  **Question or objective sufficiently described?** | **2.**  **Study design evident and approp-riate?** | **3.**  **Method of subject/ compar-ison group selection or source of inform-ation/input variables described and approp-riate?** | **4.**  **Subject (and compar-ison group, if applicable) character-istics sufficiently described?** | **5.**  **If interven-tional and random allocation was possible, was it described?** | **6.**  **If interven-tional and blinding of investi-gators was possible, was it reported?** | **7.**  **If interven-tional and blinding of subjects was possible, was it reported?** | **8.**  **Outcome and (if applicable) exposure measure(s) well defined and robust to measure-ment/mis- classification bias? Means of assessment reported?** | **9.**  **Sample size approp-riate?** | **10.**  **Analytic methods described/ justified and approp-riate?** | **11.**  **Some estimate of variance is reported for the main results?** | **12.**  **Controlled for confound-ing?** | **13.**  **Results reported in sufficient detail?** | **14.**  **Conclu-sions supported by the results?** | **Total** | **Summary score (%)** |
| --- | --- | --- | --- | --- | --- | --- | --- | --- | --- | --- | --- | --- | --- | --- | --- | --- |
| Beuchat et al., 2005 | YES | YES | NO | NO | PARTIAL | N/A | N/A | YES | PARTIAL | YES | PARTIAL | PARTIAL | PARTIAL | PARTIAL | **14/24** | **58%** |
| Block et al., 2016 | PARTIAL | PARTIAL | PARTIAL | NO | N/A | N/A | N/A | NO | PARTIAL | NO | NO | N/A | NO | PARTIAL | **5/20** | **25%** |
| Drake-Brockman et al., 2016 | YES | YES | YES | PARTIAL | N/A | N/A | N/A | YES | YES | PARTIAL | N/A | N/A | PARTIAL | YES | **15/18** | **83%** |
| Iqbal et al., 2016 | YES | PARTIAL | PARTIAL | YES | NO | N/A | N/A | YES | YES | NO | PARTIAL | NO | YES | YES | **15/24** | **63%** |
| Liebert et al., 2016 | YES | YES | YES | YES | PARTIAL | N/A | N/A | YES | YES | YES | YES | PARTIAL | YES | YES | **22/24** | **92%** |
| Liu et al., 2009; 2010 | YES | YES | PARTIAL | PARTIAL | YES | N/A | N/A | YES | YES | YES | YES | PARTIAL | YES | YES | **21/24** | **88%** |
| Liu et al., 2009a (Exp. 1) | YES | YES | PARTIAL | PARTIAL | NO | N/A | N/A | YES | PARTIAL | YES | YES | PARTIAL | YES | YES | **18/24** | **75%** |
| Liu et al., 2009a (Exp. 2) | PARTIAL | YES | PARTIAL | PARTIAL | NO | N/A | N/A | YES | YES | YES | YES | PARTIAL | YES | YES | **18/24** | **75%** |
| Ormerod et al., 2002 | YES | PARTIAL | NO | NO | NO | N/A | N/A | PARTIAL | NO | NO | NO | PARTIAL | NO | YES | **7/24** | **29%** |
| Sanderson et al., 2008 | YES | YES | YES | YES | NO | N/A | N/A | YES | YES | YES | PARTIAL | PARTIAL | YES | YES | **20/24** | **83%** |
| Schaer et al., 2015 | YES | YES | PARTIAL | YES | PARTIAL | N/A | N/A | YES | YES | NO | NO | PARTIAL | YES | YES | **17/24** | **71%** |
| Schlosser et al., 2019 | YES | YES | PARTIAL | YES | YES | N/A | N/A | YES | YES | YES | YES | PARTIAL | YES | YES | **22/24** | **92%** |
| Via et al., 2002 | YES | YES | PARTIAL | NO | N/A | N/A | N/A | PARTIAL | YES | PARTIAL | NO | PARTIAL | PARTIAL | YES | **13/22** | **59%** |

# Table S7: Summary of clinical, surrogate, and process outcomes for each study

| STUDY | CLINICAL OUTCOMES | | | | SURROGATE OUTCOMES | | | | | | | | | PROCESS OUTCOMES | | | | | QUALITY RATING |
| --- | --- | --- | --- | --- | --- | --- | --- | --- | --- | --- | --- | --- | --- | --- | --- | --- | --- | --- | --- |
|  | Reduced patient mortality | Reduced patient morbidity | | Fewer complications | Earlier identification of deterioration | Reduced number or duration of adverse events | Decreased missed vital sign changes | Reduced time to detect vital sign changes | Increased alarms detected | Reduced time to detect alarms | Increased unexpected events detected | Reduced time to detect unexpected events | Improved situation awareness | Changed patterns of gaze behaviour | Increased time focused on patient | Changed time to do other tasks | Improve information sharing | Clinician opinions |  |
|  | Clinical | | | | Vital sign changes | | | | Alarms | | Unexpected events | | Situation awareness | Gaze behaviour | | Time for other tasks | Information sharing | Clinician opinions |  |
| Beuchat et al., 2005 |  |  |  | |  |  | **+** | **+** |  |  |  |  |  | **+** | **+** |  |  |  | 58% |
| Block et al., 1995 |  |  |  | |  |  |  |  |  |  |  |  |  |  |  |  |  | **+** | 25% |
| Drake-Brockman et al., 2016 |  |  |  | |  |  |  |  |  |  |  |  |  |  |  |  |  | **+** | 83% |
| Iqbal et al., 2016 |  |  |  | |  |  |  | **+** |  |  |  |  |  |  |  | • |  | **+** | 63% |
| Liebert et al., 2016 |  |  |  | | • |  |  |  |  |  |  |  |  | **+** | **+** |  |  | **+** | 92% |
| Liu et al., 2009; 2010 |  |  |  | |  |  |  |  |  |  |  |  |  | **+** • | **+** |  |  | • | 73% |
| Liu et al., 2009 (Exp. 1) |  |  |  | |  |  |  |  |  |  | • | • |  | **+** | **+** |  |  | • | 75% |
| Liu et al., 2009 (Exp. 2) |  |  |  | |  |  |  |  |  |  | **+** • | **+** • |  | **+** | **+** |  |  | **+** | 75% |
| Ormerod et al., 2002 |  |  |  | |  |  |  |  |  |  |  |  |  | **+** | **+** | **+** |  | **+** | 29% |
| Sanderson et al., 2008 |  |  |  | |  |  |  |  |  |  | • | • |  |  |  |  |  | **+** | 83% |
| Schaer et al., 2015 |  |  |  | |  |  |  |  |  |  |  |  |  |  |  |  |  | **+** | 71% |
| Schlosser et al., 2019 |  |  |  | |  |  |  |  | **+** | • |  |  |  |  |  |  |  | **+ −** | 92%/75% |
| Via et al., 2002 |  |  |  | |  |  |  |  |  |  |  |  |  |  |  |  |  | **+** | 59% |
| Vorraber et al., 2014 |  |  |  | |  |  |  |  |  |  |  |  |  | **+** |  |  |  | **+** | 30% |
| Yoshida et al., 2014 |  |  |  | |  |  |  |  |  |  |  |  |  |  |  |  |  | **+** | 39% |

**+** = positive effect of HWD; **−** = negative effect of HWD; • = no difference between HWD and other condition; (ns) = non-significant trend in indicated direction. If a study assessed more than one outcome in each column, multiple symbols are shown.
